# Development and preliminary validation of a hybrid three-dimension printed model for glaucoma drainage device surgery training: a prospective simulation-based study

**DOI:** 10.1186/s12886-026-05034-x

**Published:** 2026-06-30

**Authors:** Adi Mohammed Al Owaifeer, Ahmed Al-Yahya, Ahmed Mashary Allubly, Khalid Ali Alalmaee, Anas Alqurashi, Abdulrahman AlKaff, Sami AlShahwan

**Affiliations:** 1 Glaucoma Division, King Khaled Eye Specialist Hospital and Research Center, Riyadh, Saudi Arabia; 2 Ophthalmology Unit, Department of Surgery, College of Medicine, King Faisal University, P.O. Box 400, Al-Ahsa, 31982 Saudi Arabia; 3 Anesthesia Department, King Fahad Medical City, Riyadh, Saudi Arabia; 4 Rehabilitation Department, Prince Sultan Military Medical City, Riyadh, Saudi Arabia; 5 College of Medicine, Umm Alqura University, Makah, Saudi Arabia; 6 Research Department, King Khaled Eye Specialist Hospital and Research Center, Riyadh, Saudi Arabia

**Keywords:** Three-dimensional printing, Computer-aided design, Glaucoma drainage device surgery, Surgical simulation, Training, Preliminary validation

## Abstract

**Background:**

We aimed to develop a three-dimensional **(**3D) printed glaucoma drainage device (GDD) surgery model and evaluate its realism and procedural performance compared with a commercially available GDD.

**Methods:**

This prospective, simulation-based pilot feasibility study assessed preliminary face and content validity evidence for a 3D-printed GDD model. Fourteen glaucoma specialists (eight fellows and six consultants) were recruited using a convenience sample from a single tertiary eye hospital. Participants performed two simulated GDD surgeries on ovine eyes in a surgical training wet lab, first using the 3D-printed model and then using a commercially available Baerveldt glaucoma implant (BGI). Procedure time was measured using a stopwatch for comparative analysis, and participants evaluated technical realism, visual realism, and overall utility relative to the BGI on a 5-point Likert scale (1 = not at all similar/not useful at all to 5 = extremely similar/extremely useful). The study was non-randomized, and participant blinding was not feasible because of visible differences between the devices.

**Results:**

Total procedure duration was longer for the 3D-printed model than for the BGI (median interquartile range [IQR], 530.5 [302.5] vs. 342 [132.5] s; *p* < 0.001), with significantly longer times for plate insertion (*p* = 0.039), tube insertion (*p* = 0.001), and tube fixation (*p* = 0.025), except for plate fixation (*p* = 0.078). Technical realism was rated highest for plate and tube fixation, with median [IQR] scores of 4.00 [1] and 4.00 [0], respectively, and overall model utility was rated 4.00 [1]. When comparing consultants and glaucoma fellows, only BGI plate fixation time differed significantly (112.5 [101] vs. 180 [94] s; *p* = 0.023).

**Conclusions:**

Although procedure times were longer with the 3D-printed model than with the commercial implant, the model demonstrated acceptable moderate-to-high ratings for realism and high overall utility, supporting its potential role as a feasible simulation-based training tool for GDD surgery.

**Supplementary Information:**

The online version contains supplementary material available at 10.1186/s12886-026-05034-x.

## Background

Glaucoma drainage devices (GDDs) are among the surgical modalities used in managing glaucoma, a progressive optic neuropathy related to elevated intraocular pressure (IOP) [1]. The concept behind GDD surgery is to insert a tube into the eye that is connected to a plate sutured externally to the ocular wall (sclera). The implanted GDD acts as a filtration pathway that directs aqueous humor out of the eye, thereby lowering the IOP.

Surgical wet-lab training for glaucoma fellows and residents is an effective way to prepare trainees before operating in actual surgical settings with patients [2]. Previously published training models cover various ophthalmic procedures, such as cataract extraction [3], trabeculectomy [4], and strabismus surgery [5]. However, resources for developing and establishing GDD training models are limited despite the steep learning curve associated with these procedures, especially at the beginning, due to the technical complexity of plate manipulation and tube insertion and the limited case volumes available to trainees. A previous report described the use of an actual GDD, the Ahmed Glaucoma Valve^®^ (New World Medical, Inc., Rancho Cucamonga, CA), on freshly enucleated pig eyes for training [6]. However, acquiring commercial devices for practice is expensive and may not be feasible in all settings.

Three-dimensional (3D) printing is a revolutionary technology that allows the fabrication of physical objects by adding layers of different materials in a 3D manner, enabling low-cost reproduction. This technology has been applied to surgical training models in various medical specialties [7]. However, accessible and reproducible 3D-printed models for GDD surgeries are limited, and existing wet-lab training mainly relies on commercial implants that may be costly, single-use, and difficult to obtain for training. Therefore, this study utilized 3D printing to develop a GDD model and compare it with a commercially available implant based on procedural time, surgical step handling (technical realism), appearance (visual realism), and overall training utility in an ovine (sheep) eye model.

## Methods

This prospective, wet-lab, simulation-based pilot study involved the development and preliminary evaluation of face and content validity of a 3D-printed model for training purposes in a surgical wet lab using sheep eyes. It was conducted at a single, specialized tertiary eye hospital and research center. The study was performed in accordance with the Declaration of Helsinki. Ethical approval was obtained from the hospital’s Institutional Review Board (IRB No. 20149-P), and written informed consent was obtained from all participants. Fresh sheep eyes were obtained from a local abattoir for wet-lab teaching. The eyes were stored in accordance with the institutional policies. No animals were euthanized for research or teaching purposes.

### Model design

The GDD selected for simulation in this study was the Baerveldt^®^ glaucoma implant (BGI) (Abbott Medical Optics, Santa Ana, CA). The model consisted of two parts: a plate and a tube. We created a 3D model of the plate using SolidWorks CAD software *(*SolidWorks Corp, Waltham, MA), to approximate the general size of the BG 101–350 model with a surface area of 350 mm^2^. Adjustments were made by upscaling the model by a factor of 1.3, as sheep eyes are approximately 30% larger than human eyes [8]. The plate was then printed using a fused deposition modeling (FDM) printer (i3 MK3, Prusa Research, Prague, Czech Republic), which uses thermoplastic polymers in filament form to create 3D objects. The filament used was white polylactic acid (PLA), with a diameter of 1.75 mm, and the layer thickness was set at 0.10 mm. The next step was to thermally attach 32-mm-long peripheral intravenous catheter (PIVC) tubes to the anterior surfaces of the printed plates in an orientation that simulated the tube position used during GDD surgery. No additional assembly steps were required beyond attachment of the tube to the printed plate. In this prototype, the tube was thermally attached; however, other attachment methods, such as adhesive fixation, may also be considered in future refinements. The model design was provided in STL format to allow access, modification, and further refinement for non-commercial educational use (see Additional file 1).

### Study participants

To evaluate the realism of the 3D-printed model relative to the actual GDD procedure, we recruited glaucoma fellows and senior glaucoma consultants using a convenience sample from a single tertiary eye hospital. Because this was a pilot feasibility and preliminary validation study, no formal inclusion or exclusion criteria were prespecified, and the sample size was determined by the number of voluntary participants; therefore, no power calculation was performed. To better assess the device for training purposes, all fellows were recruited at the end of their training period and had prior experience with GDD surgery as part of their 2-year fellowship, having completed 15–25 cases. The glaucoma consultants had extensive experience and reported performing an average of 50–75 GDD implantations per year, with clinical experience ranging from 5 to 30 years. None of the participants had prior exposure to simulation-based GDD training.

Each participant performed the procedure twice consecutively on two different sheep eyes, which were inspected and selected for comparable and good tissue condition before the procedures. All participants followed a fixed sequence, starting with the 3D-printed model and then the BGI. Procedure time was measured using a stopwatch by the same observer for all participants. For each step, the participant began only after instruction from the observer, at which point the stopwatch was started. The timed steps included plate insertion, plate fixation, tube insertion, and tube fixation. Steps such as conjunctival peritomy and conjunctival closure were not measured. Additionally, participants were not blinded because the devices were visually distinguishable under the microscope due to differences in device color and tube appearance.

### Procedural details

**Fig. 1:**
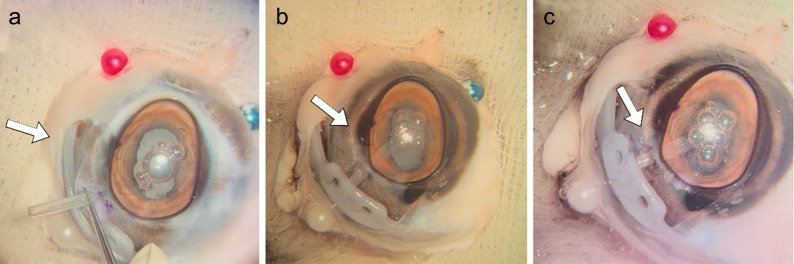
Microscopic photographs of the 3D-printed GDD model on an ovine eye. (**a**) Insertion of the three-dimensional (3D)-printed glaucoma drainage device (GDD) plate into the subconjunctival pocket (arrow). (**b**) Insertion of the tube through the sclerotomy into the anterior chamber (arrow). (**c**) Final appearance of the 3D-printed GDD after tube fixation to the sclera with sutures (arrow)

After preparation of the sheep eyes in the wet lab under the surgical microscope, the procedure began with a conjunctival peritomy in the superotemporal quadrant. The 3D-printed GDD was then inserted into the created pocket, followed by fixation of the plate to the sclera using a 9 − 0 nylon suture (Fig. 1). Once the plate was secured, the tube was trimmed to an appropriate length and inserted into the anterior chamber through a sclerotomy. The tube was then secured to the sclera with a 9 − 0 nylon suture and covered with a patch graft. Finally, conjunctival closure was performed with 8 − 0 Vicryl sutures. The same steps were repeated on a different sheep eye for the BGI.

### Model evaluation

Model evaluation was divided into two components. First, we measured the time each participant took to complete the four key steps of GDD surgery (plate insertion, plate fixation, tube insertion, and tube fixation) using both the 3D-printed model and the BGI. Second, after completing both procedures, each participant completed a questionnaire designed by the authors specifically for this simulation study. The instrument underwent face validation through independent review by two glaucoma consultants and a senior researcher, who evaluated the questionnaire’s clarity in relation to the study objectives (see Additional file 2). No formal psychometric validation was performed because of the modest sample size in this pilot study.

The questionnaire consisted of three domains. The first domain evaluated procedural handling and surgical steps or the technical realism of the 3D-printed model, in direct comparison with the BGI, across the four aforementioned steps as perceived by participants following completion of both procedures. The second domain assessed the visual realism or the general appearance of the 3D-printed model for both the plate and tube separately. The third domain was a global rating scale, evaluating the utility of the 3D-printed model as a tool for GDD surgery training. Responses were recorded on a 5-point Likert scale (1 = not at all similar/not useful at all to 5 = extremely similar/extremely useful).

### Model cost

The total material cost for the model was approximately USD 5, including the extruded PLA filament and the attached PIVC tube. The 3D printer used in this study retailed for USD 999 at the time of the study. The costs of 3D design, electricity, assembly, software, repeated production, print troubleshooting, and maintenance were not accounted for. In our study, these tasks were handled in-house by one of the authors, who is experienced in CAD software and 3D design.

### Statistical analysis

The data collection sheet was entered into an electronic database and analyzed using IBM Statistical Package for the Social Sciences Statistics version 27. The distribution of continuous variables was assessed using the Shapiro–Wilk test and by reviewing histograms and Q–Q plots. Categorical variables were presented as frequencies and percentages, whereas continuous variables were summarized as medians and interquartile ranges [IQRs]. As the procedural step durations were not normally distributed, the Wilcoxon signed-rank test was used to compare procedural step durations and total procedure duration between the 3D-printed model and the BGI, and exact two-tailed p-values were reported.

Comparisons of procedure durations between consultants and glaucoma fellows were performed using the Mann–Whitney U test, as the groups were independent. Realism and utility ratings were summarized descriptively as means with standard deviations (SDs) and medians with IQRs and were not subjected to inferential statistical testing. Effect sizes (r) were calculated from the standardized Z statistic for the Wilcoxon signed-rank and Mann–Whitney U tests. The overall technical realism score for each participant was calculated as the mean of the four technical realism steps. A two-sided p-value < 0.05 was considered statistically significant.

### Patient and public involvement

Patients and/or the public were not involved in the conception, design, conduct, or reporting of this simulation wet-lab study, which recruited trainee surgeons rather than patient participants. The questionnaire was reviewed by expert clinicians for content and clarity; this review did not involve patients or members of the public. Participant feedback was collected as study data and did not influence recruitment, outcomes, or study conduct.

## Results

### Participant characteristics

Fourteen glaucoma specialists participated in the study: 8 (57.1%) male and 6 (42.9%) female, including 8 (57.1%) glaucoma fellows and 6 (42.9%) consultants (Table 1).

**Table 1:** Baseline characteristics of the study participants

Variable	*N* (14)	Percentage
**Sex**		
Male	8	57.1
Female	6	42.9
**Level**		
Fellow	8	57.1
Consultant	6	42.9

### Procedural time outcomes

**Fig. 2:**
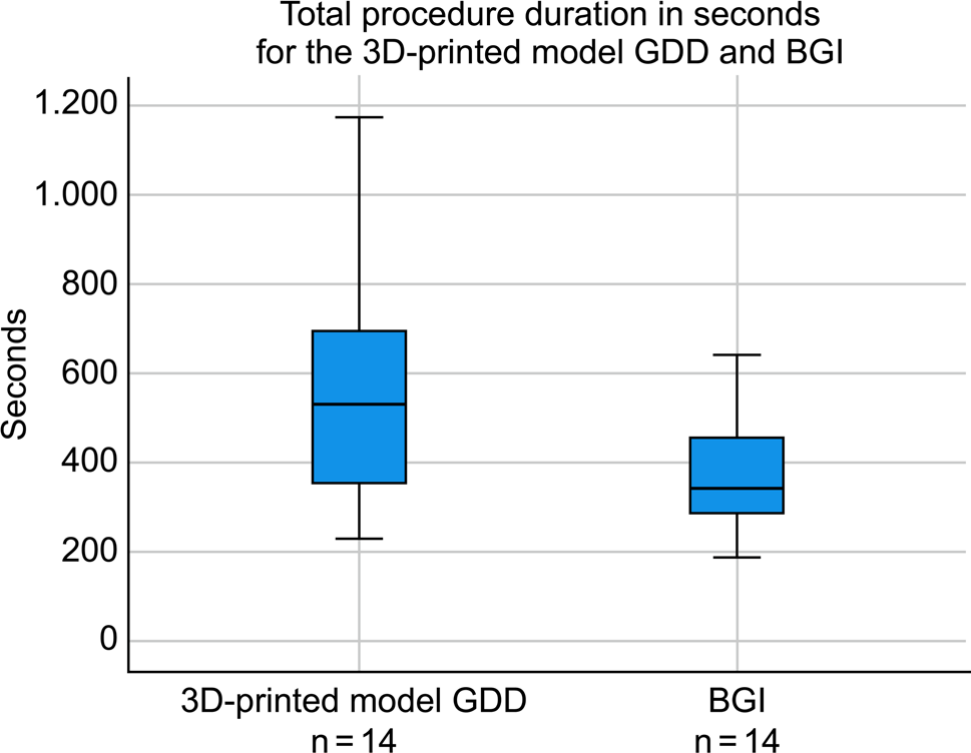
Box plot of total procedure duration for the 3D-printed GDD model and the BGI. GDD, glaucoma drainage device; BGI, Baerveldt glaucoma implant

The total procedure duration was significantly longer for the 3D-printed model than for the BGI (median [IQR], 530.5 [302.5] vs. 342 [132.5] s; *p* < 0.001), with a large effect size (*r* = 0.86). Procedural step durations were also significantly longer for plate insertion (58 [14] vs. 50 [14] s; *p* = 0.039; *r* = 0.55), tube insertion (168 [144] vs. 41 [23] s; *p* = 0.001; *r* = 0.81), and tube fixation (122.5 [64.5] vs. 94 [43] s; *p* = 0.025; *r* = 0.60). The difference in plate fixation time was not statistically significant (185.5 [99] vs. 170 [44] s; *p* = 0.078; *r* = 0.48) (Table 2; Fig. 2).

**Table 2:** Comparison of procedural step durations between the 3D-printed GDD model and BGI in 14 participants

	BGI median [IQR]	3D-printed GDD median [IQR]	*p*-value	Effect size
Plate insertion	50 [14]	58 [14]	0.039*	0.55
Plate fixation	170 [44]	185.5 [99]	0.078	0.48
Tube insertion	41 [23]	168 [144]	0.001*	0.81
Tube fixation	94 [43]	122.5 [64.5]	0.025*	0.60
Total	342 [132.5]	530.5 [302.5]	< 0.001*	0.86

Times are presented in seconds as median [IQR]. Exact p-values were calculated using the Wilcoxon signed-rank test. *Indicates statistical significance at *p* < 0.05. Effect size was calculated from the standardized Z statistic for the Wilcoxon signed-rank test

3D = three-dimensional; GDD = glaucoma drainage device; BGI = Baerveldt glaucoma implant

### Technical and visual realism and model utility

**Fig. 3:**
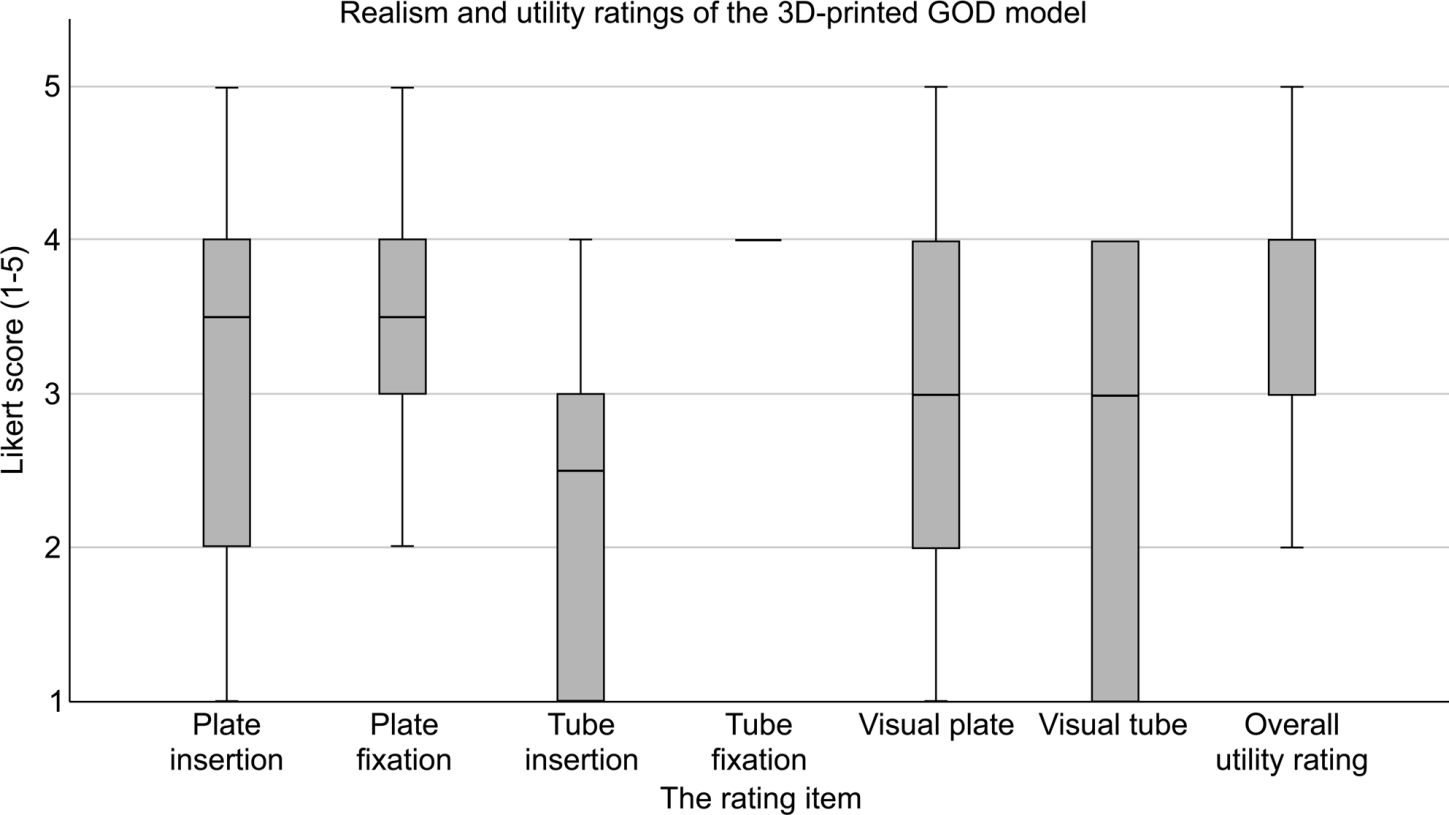
Box plots of realism and utility ratings for the 3D-printed GDD compared with the BGI. Rated by 14 participants. Boxes indicate the median and interquartile range; 1 = lowest, 5 = highest. GDD, glaucoma drainage device; BGI, Baerveldt glaucoma implant

In the technical realism domain, plate and tube fixation received the highest ratings, with median [IQR] scores of 4.00 [1] and 4.00 [0], respectively, followed by plate insertion (3.50 [2]), whereas tube insertion received the lowest rating (2.50 [2]). The overall technical rating was 3.50 [1]. For visual realism, the plate and tube were rated 3.00 [2] and 3.00 [3], respectively. The overall model utility score was 4.00 [1] (Table 3; Fig. 3).

**Table 3:** Realism and utility ratings of the 3D-printed GDD model compared with the BGI

	Realism Score: 1–5	
	Mean (SD)	Median [IQR]
Technical		
Plate insertion	3.21 (1.12)	3.50 [2]
Plate fixation	3.86 (0.86)	4.00 [1]
Tube insertion	2.21 (1.19)	2.50 [2]
Tube fixation	3.86 (0.77)	4.00 [0]
Overall technical rating	3.29 (0.63)	3.50 [1]
Visual		
Plate	3.07 (1.27)	3.00 [2]
Tube	2.64 (1.39)	3.00 [3]
Overall model utility		
Score	3.71 (0.91)	4.00 [1]

Scores are on a 5-point Likert scale (1 = lowest, 5 = highest) and are presented as mean (SD) and median [IQR]. The overall technical rating represents the mean of the four technical realism items

3D = three-dimensional; GDD = glaucoma drainage device; BGI = Baerveldt glaucoma implant

### Comparison between consultants and glaucoma fellows

When comparing consultants and glaucoma fellows, a statistically significant difference was observed only for the BGI plate fixation step, with consultants being faster than fellows (median [IQR] 112.5 [101] vs. 180 [94] s; *p* = 0.023; *r* = 0.61). No statistically significant differences were observed for the remaining procedural steps (Table 4).

**Table 4:** Comparison of procedural step durations between consultants and fellows for the 3D-printed GDD and BGI

Procedure steps	Group	Median [IQR]	U	*p*-value	Effect size
Plate insertion					
3D-printed GDD	Consultant	58 [39]	22.5	0.846	0.05
	Glaucoma fellow	55.5 [21]			
BGI	Consultant	48 [29]	18.0	0.437	0.21
	Glaucoma fellow	50 [64]			
Plate fixation					
3D-printed GDD	Consultant	148.5 [202]	12.0	0.121	0.41
	Glaucoma fellow	205.5 [143]			
BGI	Consultant	112.5 [101]	6.5	0.023*	0.61
	Glaucoma fellow	180 [94]			
Tube insertion					
3D-printed GDD	Consultant	75 [201]	12.0	0.121	0.41
	Glaucoma fellow	186 [202]			
BGI	Consultant	37 [27]	16.5	0.332	0.26
	Glaucoma fellow	46 [71]			
Tube fixation					
3D-printed GDD	Consultant	79 [88]	18.0	0.439	0.21
	Glaucoma fellow	125.5 [39]			
BGI	Consultant	72 [44]	13.0	0.155	0.38
	Glaucoma fellow	109.5[52]			

Times are presented in seconds as median [IQR]. P-values were calculated using the Mann–Whitney U test. *Indicates statistical significance at *p* < 0.05. Effect size was calculated from the standardized Z statistic for the Mann–Whitney U test. 3D = three-dimensional; GDD = glaucoma drainage device; BGI = Baerveldt glaucoma implant

## Discussion

In this study, we designed a 3D-printed GDD and demonstrated that it can be used for simulation-based GDD wet-lab training. The model provided an acceptable representation of the technical workflow, including procedure time, realism, and utility, relative to a commercially available GDD used as a reference for comparison. The model also has the added advantages of low cost, accessibility, and reproducibility.

Procedure duration was significantly longer for most steps, which may be attributable to multiple factors (Table 2). The smaller design of the BGI may have provided surgeons with greater maneuverability in a relatively larger eye, making the procedure easier and quicker to complete. Another contributing factor may have been the roughness of the 3D-printed GDD plate (made of PLA) compared with the polished, smooth surface of the BGI. A rough surface may make conjunctival and scleral handling more difficult and time-consuming. In addition, the larger tube diameter of the 3D-printed model compared with the BGI tube may have reduced flexibility and increased difficulty during insertion and fixation. As a result, significantly longer durations were observed for plate insertion, tube insertion, and tube fixation, whereas plate fixation time showed no statistically significant difference. Therefore, the use of softer materials for the plate and a 3D-printed tube may yield results more similar to those of commercial implants.

In our hybrid model, we used sheep eyes because of their resemblance to human eyes, although they are approximately 30% larger [8, 9]. To compensate for this size difference, we upscaled our 3D model accordingly. We chose to upscale the model to provide trainees with a realistic way to correlate the size of the 3D-printed GDD with sheep eyes during surgery. Future studies should assess a normal-sized 3D-printed GDD, without the 30% upscale, to achieve more accurate comparisons of procedure duration.

Despite the differences in procedure duration, most participants rated the overall usefulness for training purposes as very useful (4.00) and the technical realism as moderate to high (Table 3). This result is encouraging for the adoption of the 3D-printed model as a feasible tool to help residents and new fellows familiarize themselves with key procedural steps and acquire early skills in GDD surgery. This approach could theoretically help reduce the learning curve and assist trainees in practicing or rehearsing the steps before operating on patients, potentially resulting in shorter operative durations. However, this model does not replace the use of real GDDs in advanced courses; rather, it fills a gap by providing an accessible and reproducible training tool for wet labs.

In contrast, tube insertion received the lowest technical realism score (2.50), and visually, both the plate and tube segments received median scores of 3.00 (Table 3). This may be attributed to the method used to thermally attach the PIVC tubes to the plates and to the color difference of the plate. The BGI tube has an external diameter of approximately 0.63 mm, making fabrication of a small, flexible, and patent tube technically challenging with the 3D-printing methods available in this study. With continued advances in 3D-printing technology, it may become feasible to fabricate very small tubes using silicone, flexible resin, or other soft polymers that more closely approximate the flexibility and handling characteristics of GDD tubing.

When comparing consultants and glaucoma fellows, we observed a statistically significant difference only in the BGI plate fixation step (Table 4). Consultants completed this step faster than the glaucoma fellows, likely because plate fixation can be significantly improved with experience and repetition. Although the 3D-printed model showed a median difference of 57 s between groups, this did not reach statistical significance. The lack of significant differences in the remaining procedure steps may have been due to the small sample size. However, these findings suggest that prior skills and surgical experience influence procedure duration.

The model was evaluated by experienced surgeons, and we anticipate that its primary educational value may be for residents and novice trainees; however, future studies are needed to assess its impact in these groups and compare it with that in glaucoma fellows and consultants. In addition, future work should assess how 3D-printed devices may help reduce the steep learning curve associated with surgical ophthalmology procedures among trainees.

3D printing has been used to develop surgical training models in various medical specialties [7]. Our study aligns with prior work demonstrating the value of 3D printing for ophthalmic surgical education. An ideal training model would be completely 3D-printed; however, this was not feasible in our study, given current technological limitations. These limitations affected our model in two ways. First, we had to use PIVC tubes as a substitute for the BGI tube. Printing small-gauge, flexible tubes with patent lumens is extremely difficult with currently available retail FDM and stereolithography printers. Although flexible materials have recently emerged, maintaining lumen patency in a 3D-printed flexible tube at the small scale required to simulate a real BGI tube remains challenging.

Second, the training setup required the use of an animal eye, as it was not possible to 3D print an all-in-one training model. The eye has a complex anatomy with multiple unique soft tissues. The conjunctiva, sclera, and extraocular muscles—each critical in GDD surgery—have distinct physical characteristics. At the time of writing, no available 3D printer can produce objects with sufficient anatomical fidelity and realistic soft tissue properties to mimic these tissues [10]. Given the rapid pace of technological advancement, we hope to develop an all-in-one 3D-printed training model in the near future. Accordingly, given these limitations, we adopted a hybrid model design.

Several ophthalmic wet-lab and simulation models have been proposed for surgical training, including models for cataract surgery, trabeculectomy, strabismus surgery, and tube shunt surgery [3–6]. Plemel et al. previously described a GDD teaching model using pig eyes, in which residents practiced steps of Ahmed glaucoma valve implantation, including device anchoring, tube insertion, scleral patch graft placement, and conjunctival closure [6]. In this study, we used a 3D-printed device to simulate a commercial implant while maintaining low cost, reproducibility, and accessibility, thereby enabling more frequent practice sessions. However, the model still requires further refinement, particularly of the tube component. Porteous et al. proposed a trabeculectomy training model using only the surface of an apple to simulate the eye, whereas Jagan et al. designed a 3D-printed silicone eye model for strabismus surgery training [4, 5].

Many groups have adopted a hybrid approach in 3D-printed surgical training models. One group of maxillofacial surgeons created a training model using 3D-printed parts that simulated orbital bones, produced with selective laser sintering printers, combined with single-use silicone models that simulated orbital soft tissue. The silicone soft tissue structures were created using silicone mixtures cast in negative molds that were also 3D-printed [10]. Another group of gastroenterology endoscopic surgeons developed a hybrid model to train in the resection of ampullary adenomas. A prototype for endoscopic ampullectomy was created using computer-aided design and 3D printing of an ampullary mount and a base, to which a chicken heart was attached and inserted into a silicone stomach–duodenum model [11].

Our study has limitations that future investigations using 3D printing for ophthalmology training should consider. First, the sample size was modest but appropriate for a pilot feasibility study, and the results should be interpreted accordingly. Second, all participants followed a fixed, non-randomized sequence, with the 3D-printed model performed first, which may have introduced fatigue or order effects. Third, participant blinding was not feasible because of visible differences between the devices, which could have introduced subjective bias. Fourth, the questionnaire was reviewed by experts for clarity, relevance, and alignment with the study objectives. However, formal psychometric validation was not performed. In addition, the questionnaire assessed similarity in procedural handling and appearance but did not directly evaluate implantation difficulty or ease of use. Furthermore, objective performance metrics, such as task completion accuracy, tube positioning quality, scleral fixation quality, or validated assessment tools such as OSATS, were not included in this pilot study. Therefore, the findings should be interpreted as preliminary face and content validity evidence.

Given the low cost of the 3D-printed model and the technical challenges of GDD surgery for new glaucoma fellows, we believe our model has value as a training tool. The design we developed has been made available for non-commercial educational use by interested residents and glaucoma fellows in wet-lab training and for future refinements. As 3D-printing technology evolves, future studies should address these limitations, improve tube fabrication to enhance visual realism and overall experience, and evaluate the predictive validity of the model for improving surgical skill acquisition and performance.

## Conclusions

This study provides preliminary evidence supporting the utility of 3D printing in developing a low-cost, effective training model for GDD surgery. Participants rated the model as moderate-to-high in technical realism and moderate in visual realism. Procedure times were generally longer for the 3D-printed model than for the BGI, except for plate fixation. Larger, randomized, multicenter studies are needed to further evaluate the performance of the 3D-printed model and assess its impact on trainee outcomes. As 3D-printing technology evolves, further design refinements, particularly of the tube component, are needed to improve the model’s training utility.

## Supplementary Information

Below is the link to the electronic supplementary material.

Supplementary Material 1: File name: Additional file 1. File format: .stl. Title of data: 3D-printed glaucoma drainage device model design. Description of data: STL file of the 3D-printed glaucoma drainage device plate design

Supplementary Material 2: File name: Additional file 2. File format: .docx. Title of data: Technical and visual realism rating questionnaire. Description of data: The questionnaire was used to rate the 3D-printed glaucoma drainage device model in comparison with the Baerveldt glaucoma implant as a surgical training tool. The questionnaire included three domains: technical realism, visual realism, and overall rating

## Data Availability

The datasets used and/or analyzed during the current study are available from the corresponding author on reasonable request.

## Abbreviations

3D: Three-dimensional
BGI: Baerveldt glaucoma implant
FDM: Fused deposition modeling
GDD: Glaucoma drainage device
IOP: Intraocular pressure
IQR: Interquartile range
PIVC: Peripheral intravenous catheter
PLA: Polylactic acid
SD: Standard deviation
