# Supplementary material for: Development and preliminary validation of a hybrid three-dimension printed model for glaucoma drainage device surgery training: a prospective simulation-based study
Source: BMC Ophthalmol. 2026 Jun 30;26:369. doi: 10.1186/s12886-026-05034-x (PMC13321553; doi:10.1186/s12886-026-05034-x)
Supplement: Supplementary file 2 — Supplementary Material 2: File name: Additional file 2. File format: .docx. Title of data: Technical and visual realism rating questionnaire. Description of data: The questionnaire was used to rate the 3D-printed glaucoma drainage device model in comparison with the Baerveldt glaucoma implant as a surgical training tool. The questionnaire included three domains: technical realism, visual realism, and overall rating [file 12886_2026_5034_MOESM2_ESM.docx]

**Domain 1: Technical realism:**

**Compared with the Baerveldt glaucoma implant as a surgical training tool, please rate the 3D-printed model as follows:**

1-How similar did plate insertion feel when using the 3D-printed model?

☐1-not at all similar ☐2-slightly similar ☐3-moderately similar ☐4-very similar ☐5-extremely similar

2-How similar did plate suturing feel on the 3D-printed model?

☐1-not at all similar ☐2-slightly similar ☐3-moderately similar ☐4-very similar ☐5-extremely similar

3-How similar did tube suturing feel on the 3D-printed model?

☐1-not at all similar ☐2-slightly similar ☐3-moderately similar ☐4-very similar ☐5-extremely similar

**Domain 2: Visual realism**

1-How similar was the plate appearance compared to the Baerveldt Implant?

☐1-not at all similar ☐2-slightly similar ☐3-moderately similar ☐4-very similar ☐5-extremely similar

2-How similar was the tube appearance compared to the Baerveldt Implant?

☐1-not at all similar ☐2-slightly similar ☐3-moderately similar ☐4-very similar ☐5-extremely similar

**Domain 3: Overall rating**

1-Rate the overall usefulness of the 3D-printed model for glaucoma drainage device surgery training:

☐1-not useful at all ☐2-slightly useful ☐3-moderately useful ☐4-very useful ☐5-extremely useful
